# Case Report: Myocardial dissection caused by ruptured sinus of Valsalva aneurysm in association with a bicuspid aortic valve

**DOI:** 10.3389/fcvm.2023.1289624

**Published:** 2023-11-08

**Authors:** Xinyan Zhou, Yan Xu, Qian He, Na Tan, Jixiang Chu, Bin Liu, Yu Zhu, Chengde Liao, Yu Jiang

**Affiliations:** ^1^ Department of Radiology, Kunming Yan’an Hospital (Yan’an Hospital Affiliated to Kunming Medical University), Kunming, China; ^2^ Department of Ultrasound, Kunming Yan’an Hospital (Yan’an Hospital Affiliated to Kunming Medical University), Kunming, China; ^3^ Department of Radiology, Yunnan Cancer Hospital (The Third Affiliated Hospital of Kunming Medical University), Kunming, China; ^4^ Department of Cardiovascular Surgery, Kunming Yan’an Hospital (Yan’an Hospital Affiliated to Kunming Medical University), Kunming, China

**Keywords:** sinus of Valsalva aneurysm, bicuspid aortic valve, myocardial dissection, echocardiogram, computed tomography

## Abstract

In this report, we present a case of left-right sinus fusion in a Ruptured sinus of Valsalva aneurysm (RSVA) that perforated into the myocardium, giving rise to myocardial dissection. The existence of an anomalous bicuspid aortic valve (BAV) is contemplated as a potential etiological element in this context. Employing multimodal imaging modalities, encompassing transthoracic echocardiography and computed tomography (CT), facilitated the visualization of a dissecting hematoma situated within the myocardium subsequent to the RSVA. Following this, our patient underwent an Cabrol surgical intervention, received patch repair, and underwent mitral valve annuloplasty, during which a three-year period transpired without the occurrence of any deleterious cardiac events. In summary, this report establishes the cornerstone for the surgical intervention of RSVA, shedding light on the efficacious handling of RSVA-associated myocardial dissection. It posits that the presence of a BAV may serve as a predisposing factor to RSVA rupture, potentially elevating the susceptibility to myocardial dissection. The utilization of diverse multimodal imaging methodologies played an indispensable role in the detection of a hematoma within the myocardial tissue subsequent to the RSVA rupture. The uneventful three-year postoperative follow-up of the patient underscores the efficacy of the undertaken interventions.

## 1. Introduction

Sinus of Valsalva aneurysm (SVA) is a rare cardiac anomaly, with an incidence of approximately 0.09% (1). This is primarily attributed to structural defects existing between the aortic media and fibrous ring (2). RSVA is an uncommon complication of SVA that may rupture into the cardiac chamber or the pericardial cavity, but seldom involves myocardial tearing. Patients with a bicuspid aortic valve, characterized by a two-leaflet aortic valve anomaly, often exhibit an increased risk of ascending aortic dilation due to alterations in blood flow patterns. Existing literature suggests (3) a connection between the BAV and the development of SVA. This implies that BAV may serve as a risk factor for RSVA and could potentially be associated with the interventricular septal and myocardial intramural hematoma following SVA rupture. This case presents an instance of BAV anomaly and RSVA, resulting in an interventricular and intramural myocardial hematoma.

## 2. Case report

**Figure 1:**
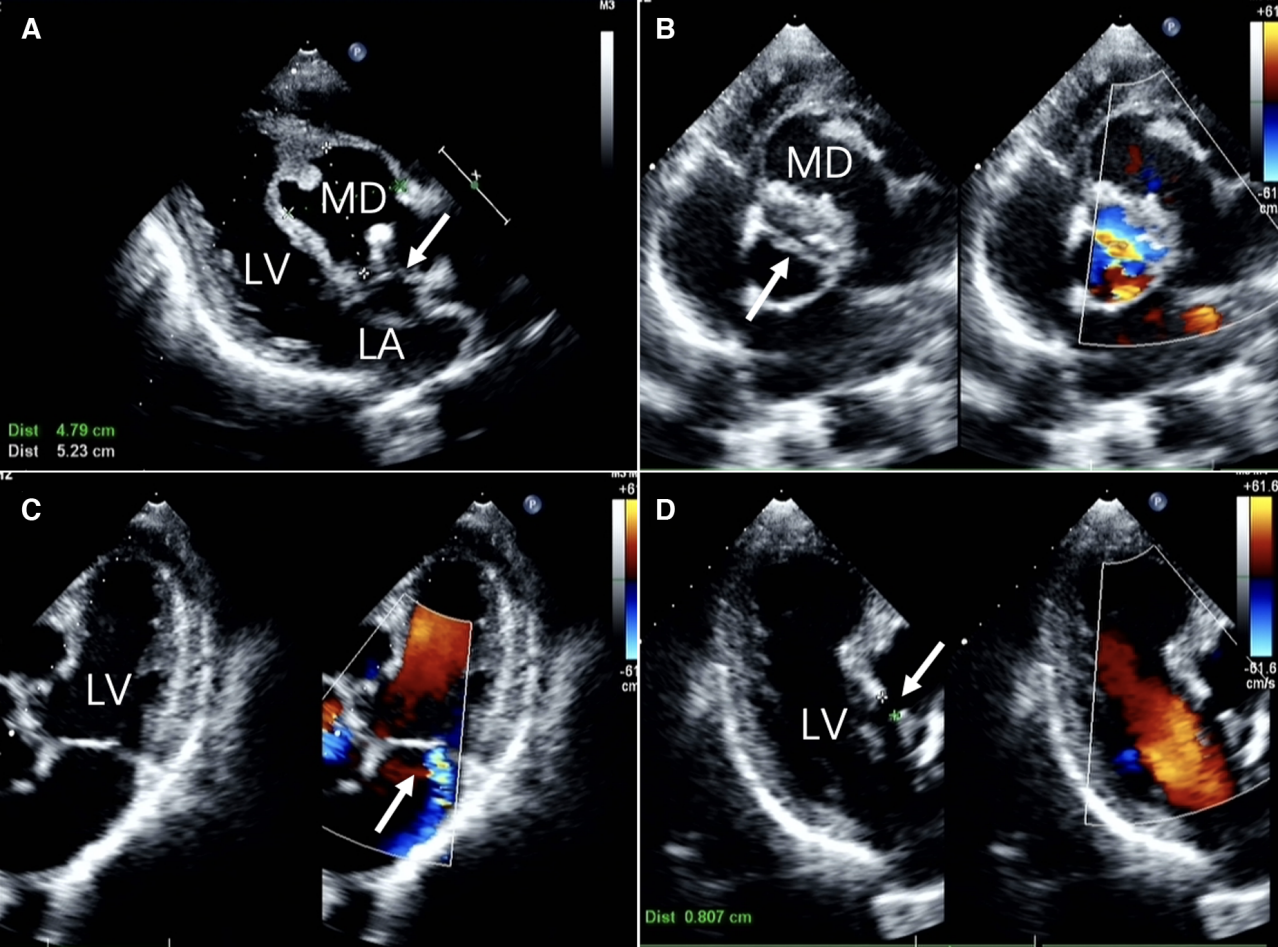
Two-dimensional transthoracic echocardiographic images (**A**) long-axis view of the left ventricle showing echo interruption, no echo in the interventricular septum.(white arrow) (**B**) the short-axis cross-sectional view of the aorta demonstrates a bicuspid aortic valve anomaly. (white arrow) (**C**) the non-standard pentachamber cardiac section reveals mitral valve eccentric regurgitation. (**D**) The three-chamber cardiac cross-section reveals the dimension of the orifice. LA, left atrium; LV, left ventricle; MD, Myocardial dissection.

**Figure 2:**
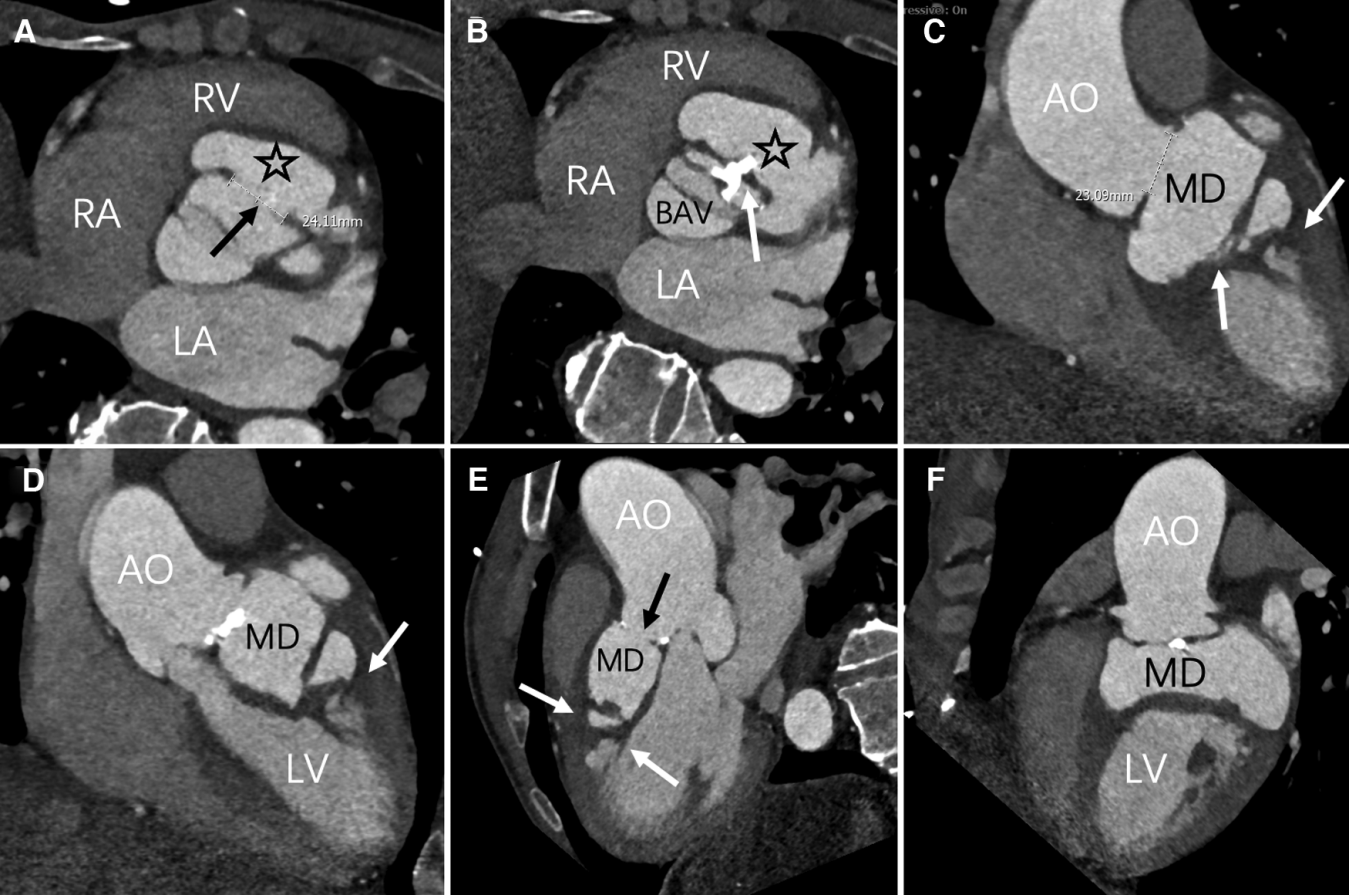
Axial position, contrast-enhanced CT imaging reveals the maximum aperture of the sinus of Valsalva aneurysm to be 2.4 cm (black arrow). The myocardial dissection originates from the fused left and right coronary sinuses (asterisk). A bicuspid aortic valve anomaly is evident, with valve leaflet thickening and calcification (white arrow) (**C**–**F**) multiPlanar Reconstruction (MPR) exhibits irregular MD, fusion of the left and right coronary sinuses (black arrow), and high-density opacities within the ventricular wall and interventricular septum (white arrow). LA, left atrium; LV, left ventricle; RA, right atrium; RV, right ventricle; AO, Aortic; MD, Myocardial dissection; BAV, bicuspid aortic valve.

A 60-year-old Chinese male presented with upper abdominal pain accompanied by dizziness and palpitations for 2 days. The patient's heart function was classified as NYHA functional class IV, and vital signs and blood biochemistry tests showed no critical indicators. Transthoracic echocardiography (TTE) revealed a myocardial dissection with a pouch-like protrusion into the right ventricle, interrupted wall echo in the septum and the basal segment of the left ventricular anterior wall, with an adherent hyperechoic structure within (Figure 1). A dual-source CT scan shows the diameter of the ascending aorta to be approximately 4.8 cm. There was a BAV and fusion of the left and right aortic sinuses, with an outward protrusion forming a cavity in the anterior aortic sinus. Myocardial dissection formation, likely due to rupture of the anterior aortic sinus, was suspected. The neck of the aneurysm had a diameter of approximately 1.4 cm, and the morphology of the aneurysm was irregular, with localized pushing on the left coronary artery (Figure 2).

After multidisciplinary consultation, the patient was diagnosed with a bicuspid aortic valve anomaly combined with a ruptured sinus of Valsalva and intramural hematoma within the interventricular septum, the basal segment of the left ventricular anterior wall, and the anterior lateral wall. Because of the patient's preexisting valve condition, we also noted the presence of moderate mitral valve regurgitation and tricuspid valve regurgitation. During our surgical exploration, we observed the formation of a partial thrombus within the heart.

**Figure 3:**
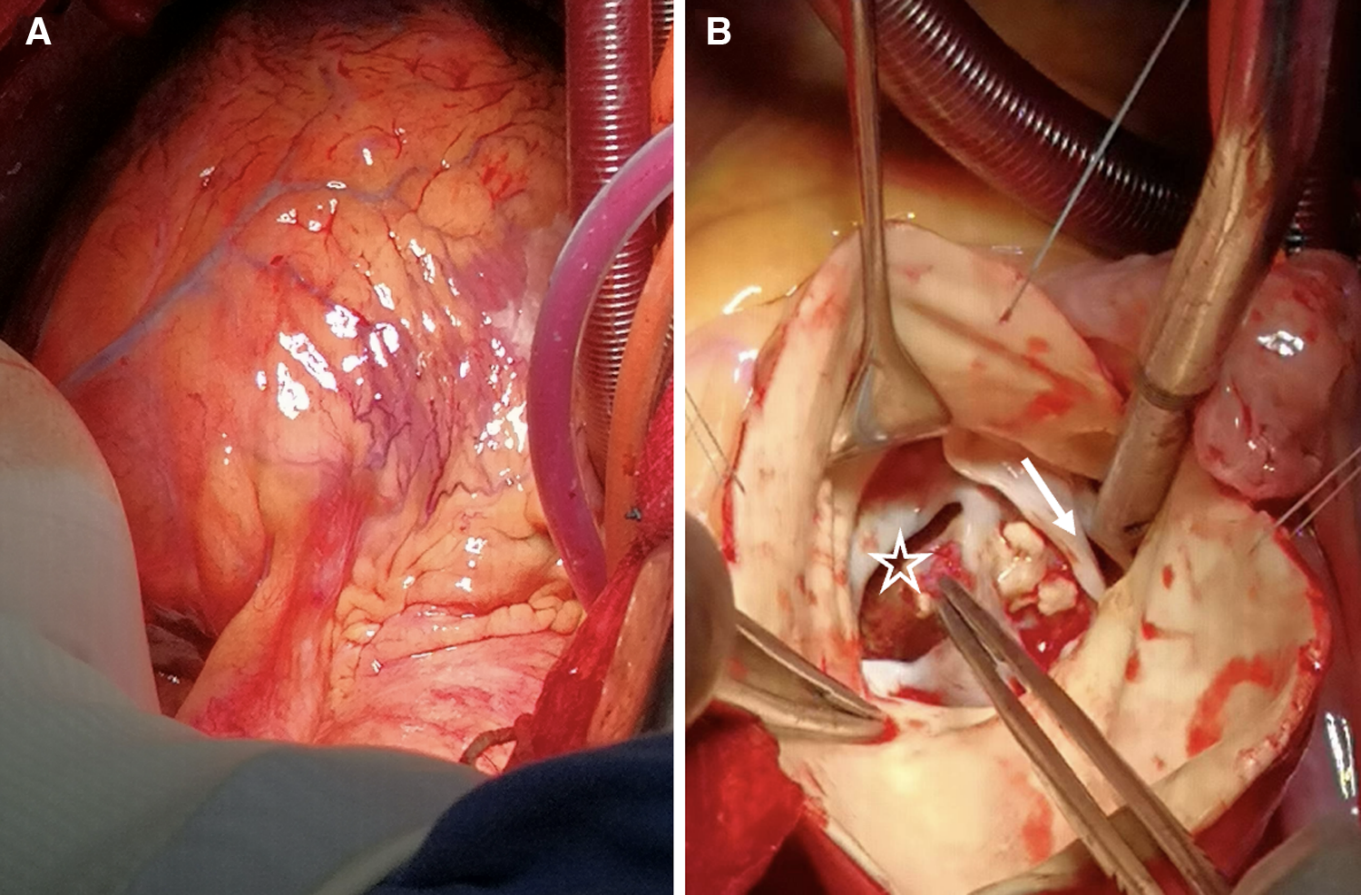
(**A**) The heart's presentation reveals evident edema. (**B**) BAV (white arrow) is discernible, accompanied by numerous calcifications on the valve leaflets. Additionally, we observe the occurrence of a sinus aneurysm rupture (asterisk), concomitant with an intraluminal thrombus.

After obtaining the patient's informed consent, surgical intervention was initiated. The procedure commenced with a midline sternotomy, revealing a dilated ascending aortic aneurysm upon opening the pericardium. Notable flutter was observed in the pulmonary artery and the conus of the right ventricle. Aortic and bicaval cannulation was performed to initiate extracorporeal circulation. Intraoperative exploration revealed left ventricular enlargement, bicuspid aortic valve deformity with anterior-posterior orientation, leaflet thickening, and calcification. A longitudinal tear of approximately 2 cm was evident in the aortic sinus, extending all the way to the interventricular septum and the posterior wall of the left ventricle, accompanied by the formation of an intracavitary thrombus (Figure 3). Following the exploration, repairs of the aortic sinus, the Cabrol procedure, and annuloplasty of the aortic valve were performed. We utilized a 21 mm mechanical valve prosthesis for the aortic valve replacement and completed interrupted sutures along the aortic annulus. Subsequently, aortic replacement was carried out using dacron tube, and approximately 0.7 cm of prosthetic material was employed for indirect coronary reimplantation in the Cabrol procedure. Subsequent transesophageal echocardiography revealed no significant regurgitation, and myocardial function was within the normal range. Closure of the atrial septum and the right atrium was performed. Partial pericardial closure was meticulously carried out to ensure hemostasis, followed by chest closure.

**Figure 4:**
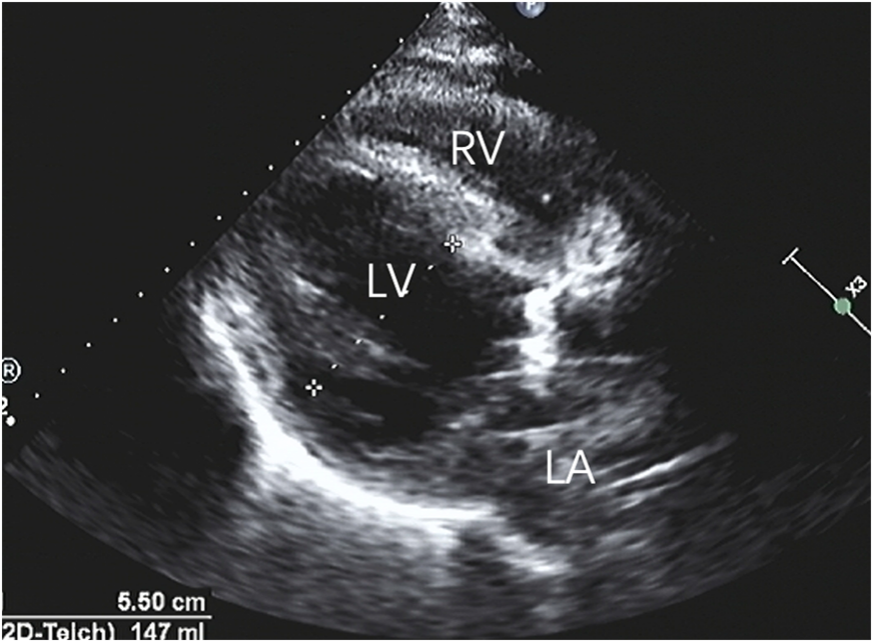
After repairing the rupture of the aortic sinus, the myocardial dissection resolved, and the anatomical structure returned to its normal state.

Postoperatively, the patient experienced a smooth recovery, with normal mechanical valve opening and closing sounds. CT scans revealed a satisfactory anastomosis of the aorta and coronary arteries, while transthoracic echocardiography (TTE) indicated a significant improvement in left ventricular ejection fraction and anterior wall motion (Figure 4). During a three-year follow-up, the patient's outcome remained favorable.

## 3. Discussion

SVA is an uncommon cardiac anomaly. According to a series of postmortem examinations (1), the incidence rate of SVA is recorded at 0.09%, with a gender ratio of 3:1 (4, 5). SVA predominantly manifests among individuals aged 30 to 45, with a heightened prevalence among individuals of Asian descent (6). The embryonic basis of SVA is linked to impaired development of the far basal part of the membranous interventricular septum. It often involves the right coronary sinus, followed by the contiguous two-thirds of the non-coronary sinus. In instances of SVA rupture, the most common occurrence is rupture into the right ventricle, followed by the right atrium (7). There exists a mere 2% likelihood of penetration into the interventricular septum and the ventricular myocardium (5). In this specific case, an Asian patient experienced SVA rupture into the interventricular septum and the left ventricular myocardium due to the fusion of the left and right coronary sinuses caused by BAV.

This International evidence-based nomenclature on the congenital bicuspid aortic valve and its aortopathy recognizes Fused type, 2-sinus type with 2 phenotypes and Partial-fusion or forme fruste (8). In this particular case, the patient presented with L-R type. BAV is associated with genetic mutations involving GATA5, NOTCH1, ACTA2, and others (9). Patients with BAV may experience fusion of the aortic sinuses, resulting in altered blood flow patterns that often lead to vascular wall stress overload. This, in turn, can lead to the loss of vascular smooth muscle cells, reduced fibrous protein content, elastic fiber rupture, and matrix disruption (10). Consequently, it could potentially increase the risk of SVA rupture and exacerbate the extent of involvement following SVA rupture. Our patient is afflicted with BAV, which renders the aortic wall vulnerable. When the SVA abruptly ruptures, the force of blood flow creates openings in the vessels and gives rise to interventricular septal defects and blood shunting. As blood continues to impact the vessel walls and endocardium, it gradually forms an intramural hematoma between the interventricular septum and the ventricular myocardium.

In a recent comprehensive review, it was determined that approximately 50% of RSVA patients manifest respiratory distress, followed by chest pain (18%). Symptoms such as palpitations, syncope, vomiting, and fever were also noted (3, 11). In this particular case, the patient presented solely with symptoms of chest pain. Interestingly, nearly 10% of patients remain asymptomatic (11). Without prompt intervention, nearly 80% of cases may progress to heart failure and sudden death, leading to a bleak prognosis for affected individuals. Hence, accurate diagnosis and timely treatment are of paramount significance.

In general, echocardiography demonstrates exceptional sensitivity to both vascular walls and myocardium. Within the ultrasound examination, our patient's presentation was characterized by an aortic sinus that protruded into the right ventricle, displaying an interrupted wall echo. Notably, there is an anomalous echo-free region in the basal segment of the interventricular septum and the left ventricular anterior wall. This manifestation signifies the rupture of the sinus aneurysm, resulting in the tearing of the corresponding interventricular septum and myocardium.

CT imaging revealed a bicuspid valve in an L-R cusp fusion in this patient, with multiple calcifications on the leaflets. The merging of the left and right coronary sinuses forms a pouch-like structure. An irregularly shaped high-density shadow appears on the interventricular septum and the left ventricular wall, indicative of a myocardial dissection within the myocardium. CT provides high-resolution three-dimensional images, which offer a more intuitive basis for clinical decision-making and complement the results of echocardiography (12).

The surgical treatment of RSVA was first reported by Lillehei and colleagues in 1957 (13). Currently, treatment is typically guided by international guidelines (14), following a similar approach as in patients with aortic root tumors. In our case, both CT scans and surgical exploration revealed myocardial dissection in the interventricular septum and the anterior wall of the left ventricle. To address these related defects and excise the aneurysm sac, thereby preventing cardiac obstruction or aortic valve dysfunction, we chose to employ bovine pericardial patch repair of the aortic sinus rupture. The literature suggests (15) that aortic valve replacement and ascending aortic replacement may provide stability to the sinus in patients with bicuspid valve anomalies and aortic root dilation. Consequently, we performed an aortic valve replacement and carried out the Cabrol procedure. Due to structural damage to the aorta, we chose the Cabrol procedure instead of the Bentall procedure.

In summary, RSVA patients with concomitant BAV often exhibit valve leaflet anomalies, necessitating meticulous exploration and complex surgical approaches for personalized repair and treatment.

## Data Availability

The original contributions presented in the study are included in the article/**Supplementary Material**, further inquiries can be directed to the corresponding authors.
