# Supplementary figures and images for: Case Report: Myocardial dissection caused by ruptured sinus of Valsalva aneurysm in association with a bicuspid aortic valve
Source: Front Cardiovasc Med. 2023 Nov 8;10:1289624. doi: 10.3389/fcvm.2023.1289624 (PMC10663329; doi:10.3389/fcvm.2023.1289624)

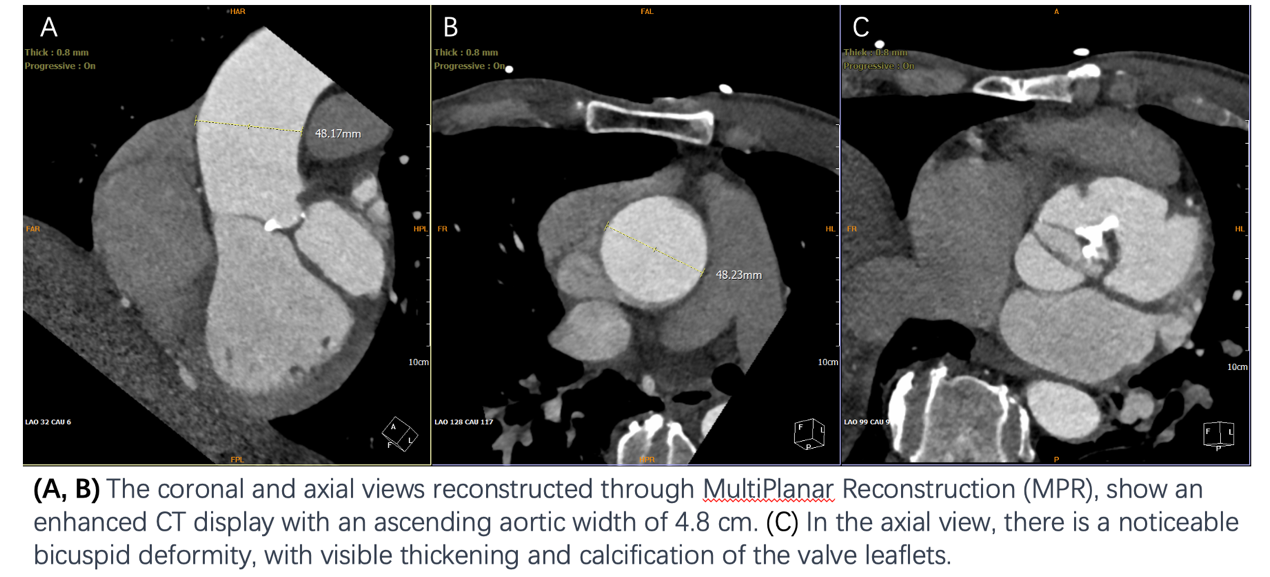


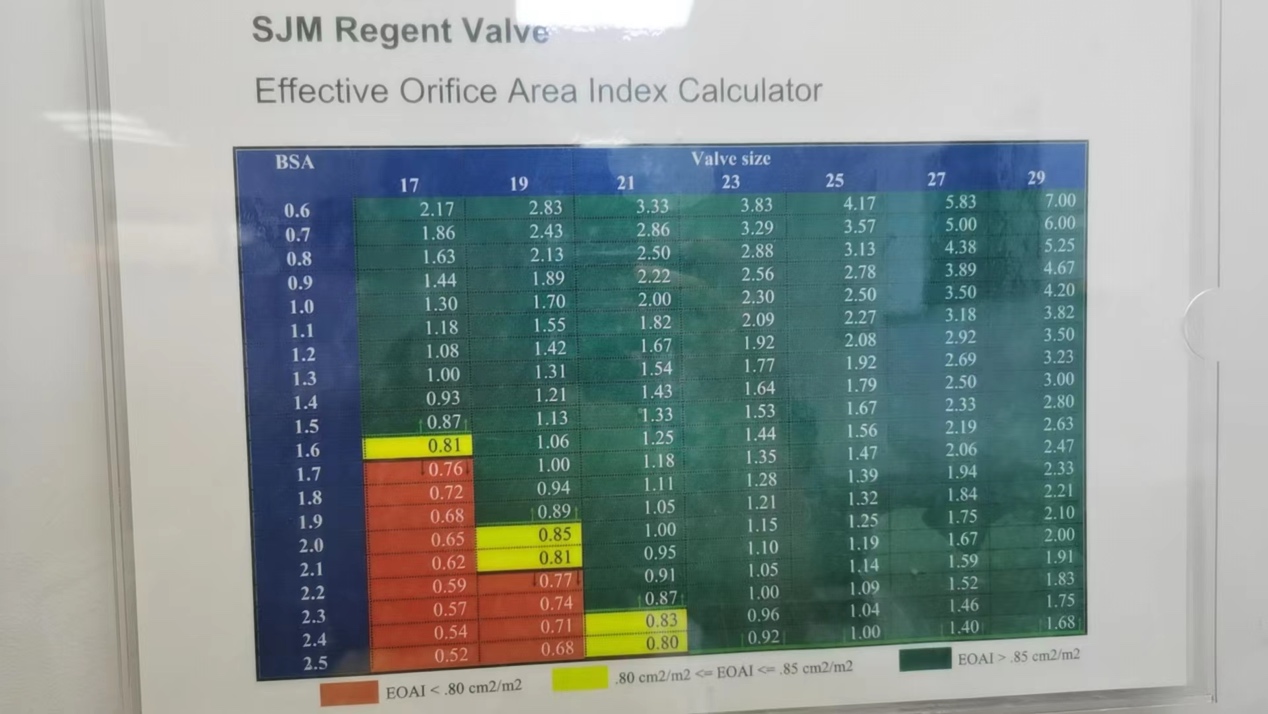

Supplement: Supplementary file 1 [file Datasheet1.docx]
